# Mucoadhesive Polymers and Their Applications in Drug Delivery Systems for the Treatment of Bladder Cancer

**DOI:** 10.3390/gels8090587

**Published:** 2022-09-15

**Authors:** Caroline S. A. de Lima, Justine P. R. O. Varca, Victória M. Alves, Kamila M. Nogueira, Cassia P. C. Cruz, M. Isabel Rial-Hermida, Sławomir S. Kadłubowski, Gustavo H. C. Varca, Ademar B. Lugão

**Affiliations:** 1 Nuclear and Energy Research Institute, IPEN-CNEN/SP—University of São Paulo, Av. Prof. Lineu Prestes, No. 2242, Cidade Universitária, São Paulo 05508-000, Brazil; 2 I+D Farma Group (GI-1645), Departamento de Farmacología, Farmacia y Tecnología Farmacéutica, Facultad de Farmacia, Instituto de Materiales (iMATUS) and Health Research Institute of Santiago de Compostela (IDIS), Universidade de Santiago de Compostela, 15782 Santiago de Compostela, Spain; 3 Institute of Applied Radiation Chemistry (IARC), Lodz University of Technology, Wroblewskiego No. 15, 93-590 Lodz, Poland

**Keywords:** mucoadhesion, drug release, bladder tumor, polymeric hydrogels, intravesical therapy

## Abstract

Bladder cancer (BC) is the tenth most common type of cancer worldwide, affecting up to four times more men than women. Depending on the stage of the tumor, different therapy protocols are applied. Non-muscle-invasive cancer englobes around 70% of the cases and is usually treated using the transurethral resection of bladder tumor (TURBIT) followed by the instillation of chemotherapy or immunotherapy. However, due to bladder anatomy and physiology, current intravesical therapies present limitations concerning permeation and time of residence. Furthermore, they require several frequent catheter insertions with a reduced interval between doses, which is highly demotivating for the patient. This scenario has encouraged several pieces of research focusing on the development of drug delivery systems (DDS) to improve drug time residence, permeation capacity, and target release. In this review, the current situation of BC is described concerning the disease and available treatments, followed by a report on the main DDS developed in the past few years, focusing on those based on mucoadhesive polymers as a strategy. A brief review of methods to evaluate mucoadhesion properties is also presented; lastly, different polymers suitable for this application are discussed.

## 1. Overview

One of the most probable causes of mortality in the worldwide population is cancer. The prevalence of this set of diseases seems to be decreasing very slowly due to enhancements in early detection and better treatments. Nevertheless, cancer remains a major problem concerning public health systems [1]. Above 18 million new cases are diagnosed each year, and one in every five people develops this condition before the age of 75 years old. Subsequently, around 10 million people die from cancer per year [2]. 

Bladder cancer is the tenth most common type, representing 3% of the new diagnoses and 2.1% of cancer deaths [2,3]. Focusing on gender, men present three to four times more chances to develop bladder cancer than women [4]. 

**Figure 1:**
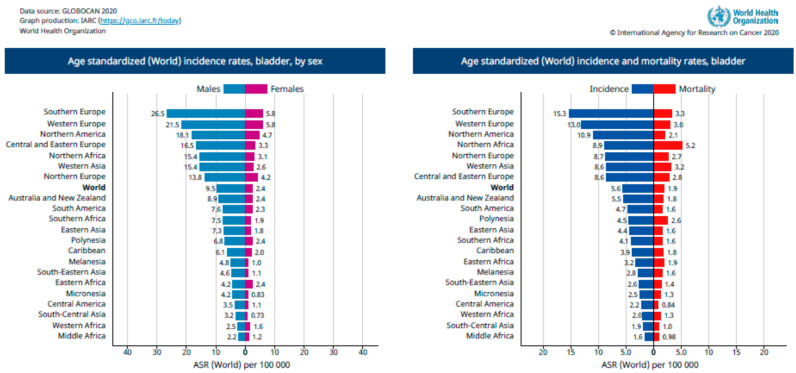
Age-standardized incidence rates of bladder cancer in the world according to the World Health Organization. Reprinted from Bladder Cancer, The Global Cancer Observatory, Copyright (2020) [2].

Moreover, there is a significant variance concerning occurrence in the geographical regions; higher rates are observed in Europe and North America, while a lower percentage of cases can be found in Latin America and Northern Africa (Figure 1). The registers concerning bladder cancer vary around the world and are more easily found in European countries and Australia. Developing countries usually lack registers of regional recurrence of cases, in addition to being deficient in providing access to care and diagnostic procedures. However, the differences in recurrence are mostly due to differences in exposure to risk factors such as cigarette smoking, chemical carcinogens, chemotherapy, pelvic radiotherapy, traces of arsenic in drinking water, or endemic chronic urinary infections caused by *Schistosoma haematobium* [3,5].

Smoking is the main risk factor for bladder cancer and is related to up to 50% of all cases, particularly urothelial tumors. On the other hand, the infection by *Schistosoma haematobium* is usually related to squamous cell carcinoma. For example, in Egypt, where there was an endemic scenario related to schistosomiasis, there is a dominance of this type of bladder cancer [3]. 

The symptoms that could potentially indicate the existence of the tumor are the presence of blood in the urine, irritative voiding symptoms such as urgency to urinate frequently, and repetitive urinary infections. Furthermore, 75% of bladder tumors are non-muscle-invasive (urothelial) and, therefore, less aggressive, while the other 25% are muscle-invasive or metastatic diseases. The stage of urothelial carcinoma is the most important prognostic factor, which is based on cytologic atypia. 

**Figure 2:**
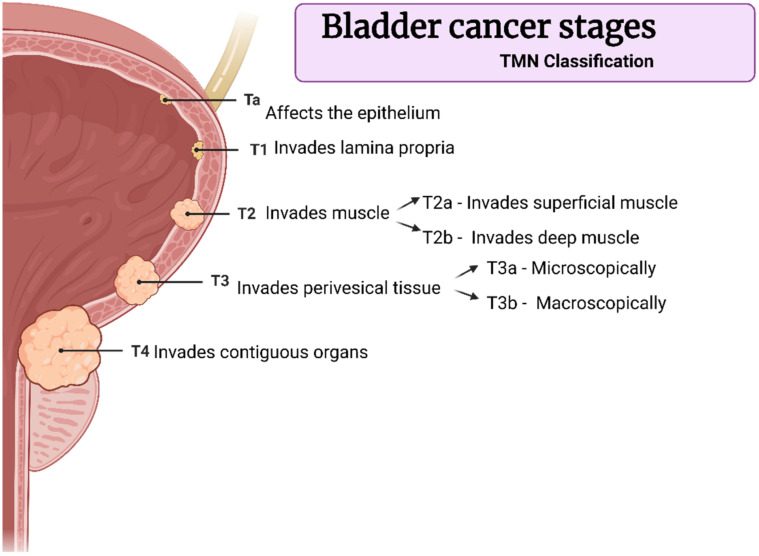
Bladder cancer stages according to TNM classification. Created with BioRender.com adapted from Surgery, 34:10, Down et al., Bladder Cancer, Pages 532–539, Copyright (2016), with permission from Elsevier [7].

The tumors are classified according to the TNM scale, which describes tumor size/depth and nodal or metastatic spread (Ta, T1, T2, T3, or T4—Figure 2), and the muscle-invasive forms are those above T2. However, T1 tumors must receive significant attention because they affect the lamina propria, which indicates their potential to become invasive [6,7]. Depending on the disease stage, different protocols of therapies are required. Superficial tumors are often treated with single instillation of mitomycin C (MMC), epirubicin, gemcitabine, or BCG (bacillus Calmette–Guérin), while more invasive tumors may demand the combination of more than one drug for chemotherapy [8,9]. 

## 2. Fundamentals 

### 2.1. Mucosa Structure

**Figure 3:**
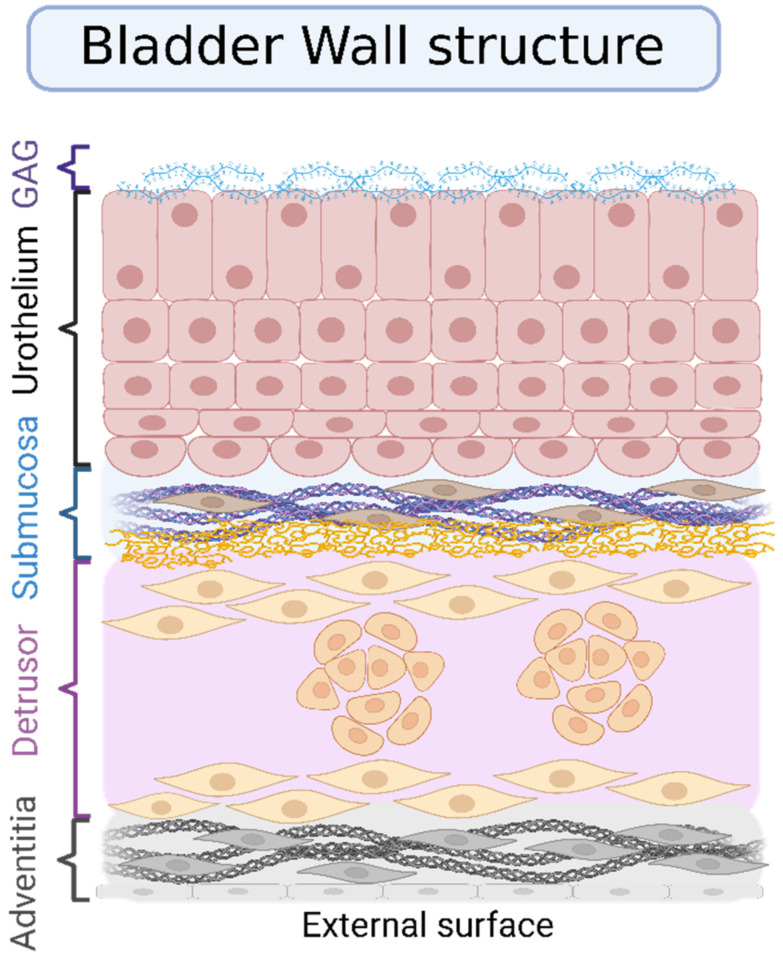
Layers that compose the bladder wall. The inner layer is called the urothelium, and its luminal surface is covered with glycosaminoglycan (GAG). The urothelium is composed of three sublayers. The first one is made of basal cells attached to a basement membrane; the superficial layers, i.e., the second and the third ones, are made of large hexagonal cells, the umbrella cells. Under the urothelium, the submucosa or lamina propria is composed of fibril-shaped or bundle-shaped collagens (type I and III), an elastin fibrous network, interstitial cells, fibroblasts, adipocytes, afferent and efferent endings, and blood vessels. Lastly, the outer layer is the muscular one (detrusor) covered by the adventitia. Created with BioRender.com, adapted from open access Wang et al., Pharmaceutics; published by MDPI, 2021 [12].

The urinary bladder wall is composed of the urothelium (inner layer), the lamina propria (submucosal connective tissue layer), the muscular layer, and the serosal layer covering it on the outer layer (Figure 3). Usually, women present 3.0 ± 1.0 mm of bladder wall thickness, while men present 3.3 ± 1.1 mm [10].

The internal face of the bladder is covered by a mucosa composed of transitional epithelium known as the urothelium, basement membrane, and sub-urothelium. The urothelium is a specialized epithelium coated with mucopolysaccharide and glycosaminoglycan, with an important function of protection from the urine. The structure of this mucosal surface is wrinkled, which allows the cycles of filling and voiding the vesicle without compromising the barrier function. The urothelium has three layers; the first one is made of basal cells attached to a basement membrane. The superficial layers, i.e., the second and the third ones, are made of large hexagonal cells, the umbrella cells. One of the main functions of the urothelium is to isolate the urine from the underlying tissues, which is possible due to the tight junctions between umbrella cells. Between the urothelium and the detrusor layer, there is a layer composed of an extracellular matrix known as the “sub-urothelium”. This layer contains fibril-shaped or bundle-shaped collagens (type I and III), an elastin fibrous network, interstitial cells, fibroblasts, adipocytes, afferent and efferent endings, blood vessels, and a muscular layer called *muscularis mucosae* [10,11]. 

### 2.2. Available Treatments

**Figure 4:**
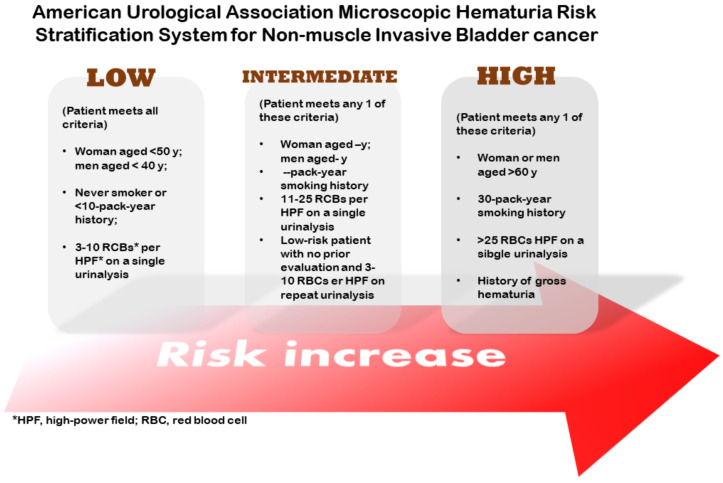
Stratification system for superficial bladder cancer.

Superficial bladder cancer is the most frequent form of bladder cancer. Usually, it is treated by telescopic removal of the tumor using a technique called transurethral resection of bladder tumor (TURBIT), followed by the instillation of chemotherapy or immunotherapy. The monitoring of the bladder is conducted by cystoscopy. Around 70% of the patients diagnosed with bladder cancer present the non-muscle-invasive kind. The American Urological Association (AUA) classifies patients into low-, intermediate-, and high-risk categories, according to factors such as age, smoking history, and symptoms (Figure 4) [9]. Low-risk tumors (low-grade Ta) are treated by TUBIT followed by a single instillation of a chemotherapy drug (MMC, epirubicin, or gemcitabine, for example) [13]. High-grade Ta and T1 just treated with TURBIT present a 50% chance of recurrence; thus, it is common to combine TURBIT with MMC or BCG. Chemotherapy is used when there is a greater risk of progression or recurrence in non-muscle-invasive types. 

When the patient presents a high risk, major surgery to remove the organ (cystectomy) could be considered. In cases where the tumor has invaded the bladder muscle, the cure may be achieved via treatment with chemotherapy, radiotherapy, or cystectomy. On the other hand, when the tumor presents too high a grade and, therefore, does not have a perspective of cure, treatment with radiotherapy and chemotherapy are extremely recommended [14]. 

BCG was developed as a vaccine for the prevention of tuberculosis disease. However, it has been applied in oncology as an immunotherapeutic for several types of cancer, including non-muscle-invasive bladder cancer. Since 1976, BCG has been recommended for superficial bladder cancer treatment for patients who present a high risk for recurrence and progression as it is the best option to delay them [6]. The vaccine acts by recruiting different types of cells in the tumor microenvironment, such as CD4^+^ and CD8^+^ lymphocytes, granulocytes, macrophages, and dendritic cells, which lead the tumor cells to apoptosis [8].

Non-metastasized bladder tumors are treated with intravesical chemotherapy. According to the tumor progression, a single chemotherapeutic or a combination may be used. Gemcitabine and cisplatin, dose-dense methotrexate, vinblastine, doxorubicin (DOX) and cisplatin (MVAC), cisplatin, methotrexate, and vinblastine (CMV), and gemcitabine and paclitaxel are the common combination drugs [8]. 

Intravesical formulations to treat bladder cancer must attend to some specific features such as having the ability to overcome the urothelium wall, molecular weight under or equal to 200 Da, pH between 6 and 7, and aqueous/organic phase partition coefficient from −0.4 to −0.2 or from −7.5 to −8.0. In addition, the presence of charge in eventual drug nanoparticles may help with cellular uptake. Positively charged particles are more rapidly absorbed into tissues in comparison with anionic or neutral particles [15,16].

Usually, the volume of drug formulation instilled into the urine bladder is around 50 mL. Afterward, micturition is prevented for 1–2 h. Regardless of the preparation of the patient, residual urine is often present in the human urinary bladder, which causes dilution of the drug or even washing it out. These circumstances demand frequent catheter insertion with a reduced interval between doses and even irritation of the urothelial lining or urinary tract infection. To overcome that, new formulations with improved mucoadhesion, targeting, and controlled delivery have been studied in the past years [15]. 

### 2.3. Drug Delivery Systems (DDS)

One of the main advantages of drug delivery systems is the release of the drug in a targeted location, increasing the absorption by the organism. Accordingly, drug delivery systems are an option to increase treatment efficacy. 

In DDS, there are generally two factors used to evaluate the efficacy of the system: the quantity of the drug loading and the duration of the presence of the drug in the organism. Consequently, the design of drug delivery systems involves the chemical formulation of the drugs, the route of administration, the form of dosage, and the use of supplemental medical devices [17].

Researchers aim to develop a target-specific, effective, and safe drug delivery system to boost therapeutic actions and reduce side effects. Advances in drug delivery studies can facilitate the development of an active carrier for targeted action with improved pharmacokinetic behavior [18].

Drug properties can vary significantly when used to treat the same symptoms, depending on the chemical composition, size, hydrophilicity, and ability to bind to a specific receptor. The drugs can suffer from insufficient bioavailability due to insolubility in physiological fluids and low permeability of different organs. Therefore, the therapeutic performance is dependent not only on the activity of the applied drug but also on the bioavailability of the targeted site [19].

The delivery of a specific drug at a programmed rate over a prolonged period is a topic of interest in the drug delivery field. The strategy of releasing drugs at slower rates is very useful for pharmaceutical ingredients that are either subject to a fast metabolism and eliminated from the organism quickly after administration or for providing extended pharmacological action. Sustained drug delivery can be reached by preventing the drug molecules from completely entering the aqueous environment for a viable period; it can be achieved by adjusting the degradation speed of the carrier or by adjusting the diffusion rate of the drug over an insoluble polymer matrix or shell. A constant dosage of drug within the therapeutic window is beneficial to counter the side-effects related, for example, to chemotherapy [20].

The ideal drug carrier should have the following characteristics: good biocompatibility and specific release of drugs at the lesion tissue or targeted cells. Even though no clinical formulation possesses all these characteristics, researchers continue to design smart DDSs with multiple functions, aiming to explore improved strategies for the treatment of diseases and to obtain promising formulations for clinical translation [21]. Below, several DDS implemented for the treatment of BC are briefly explained. 

Thiolated chitosan has been frequently explored for intravesical DDS due to its high mucoadhesive properties, but usually, this kind of system is used for the delivery of hydrophilic drugs. To allow the administration of a lipophilic drug in such a system, a self-emulsifying drug delivery system (SEDDS) decorated with thiolated chitosan was prepared. Formulations composed of S-protected chitosan complexed with sodium dodecyl sulfate (SEDDS-CS-MNA-SDS) were those able to be retained in the porcine mucosa for a longer period after voiding several times [22].

A new intravesical DDS composed of a Foley-type catheter (FT-C), which contains an inflation balloon at the tip, was developed by replacing the impermeable silicone rubber of the balloon with a permeable membrane made of interpenetrating polymer network (IPN). The system allowed the diffusion of water-soluble drugs such as MMC, providing prolonged drug release into the bladder. Drug release and anti-carcinoma cell efficacy were investigated. In vitro results showed a sustained release of MMC for up to 12 days with an inhibitory effect against HTB-9 (ATCC bladder carcinoma cell line), but that time could be extended once the drug reservoir can be reloaded without removing the catheter. In vivo short-term studies were also performed in porcine models, and the therapeutic MMC concentration was released after 2 h. However, optimization of the system and longer pre-clinical studies are needed, as little or no MMC tissue uptake was observed [23].

Kaldybekov and coworkers [24] developed maleimide-functionalized PEGylated liposomes (PED-Mal) for intravesical drug delivery. The liposomes presented good adhesion to the bladder mucosa, with a retention of 32% of the formulation after 50 min of washing. Fluorescence microscopy assays revealed that the PEGylated liposomes presented a higher capacity of permeation than conventional and PEG-Mal ones. This is mainly because the maleimide-functionalized PEG liposomes formed strong covalent bonds to the mucosa slowing down their penetration. Concerning drug release, PEG-Mal liposomes presented a sustained release through 8 h while conventional and PEGylated ones presented faster release, in 2 h and 4 h, respectively.

New approaches to drug design based on nanoparticles and nanostructures for effective drug delivery are crucial for the future of medical treatment, especially for cancer therapy. Nanotechnology associated with the appropriate material may present great potential in increasing drug delivery efficiency. Furthermore, for biomedical applications, they must be biodegradable, have a prolonged circulation half-life, not be inclined to aggregate or cause an inflammatory response in the organism, and be cost-effective. The efficacy of those structures is very dependent on their chemical properties, as well as on their size, charge, shape, surface modifications, and loading methods [25].

Nanoparticles (NPs) or nanocarriers (NCs) are increasingly considered candidates to safely carry therapeutic agents into selected sections in the body, such as a cell or a particular tissue. Various nano or micro delivery systems are designed to encapsulate the active agent such as polymeric nanocapsules, dendrimers, and liposomes. They can conceal some of the adverse biopharmaceutical properties of the molecule and replace them with properties of materials used for nano-delivery systems. Furthermore, advances in the nanomedical field are also applied for site-specific drug delivery [26]. The benefits of nanoscience and nanotechnology progress and their application in therapeutic drug delivery are huge, aiming to overcome the undesirable effects of previous therapies and develop treatments for several diseases. As a result, the pharmacokinetics can be modulated, and the transport and specific targeting through the controlled drug release with reduced dosing can be achieved. In addition, the solubility, biodistribution, and in vivo stability can be increased [25].

For instance, mesoporous silica nanoparticles were modified superficially with poly(amidoamine) (PAMAM) dendrimers through a layer-by-layer method for the delivery of DOX in the treatment of bladder cancer. The number of PAMAM dendrimer layers was capable of controlling the release rate of the system which was triggered by acid pH. The mucoadhesion was increased by enhancing the number of amino groups in the PAMAM. This was concluded after observing the increase in the hydrodynamic size of nanoparticles after electrostatic interactions between their positive charges and the mucin’s negative charges. However, there was no significant difference between two and three layers of the polymer, indicating its biding saturation [27].

**Figure 5:**
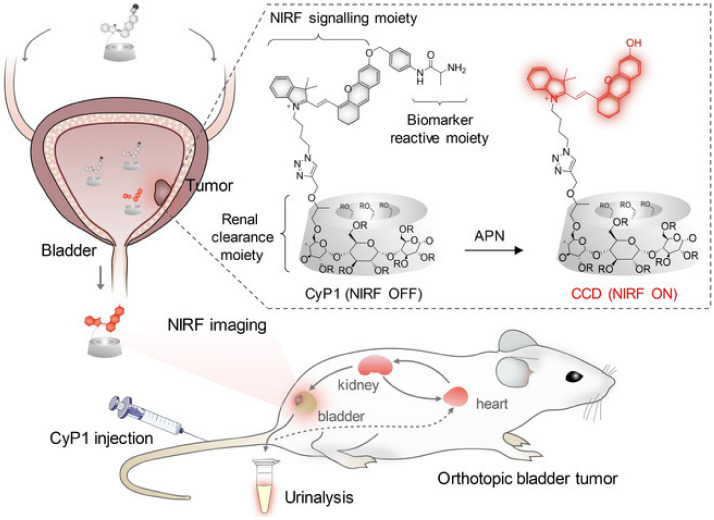
Scheme representing the design and mechanisms of the renal-clearable macromolecular reporter (CyP1) for NIRF imaging and urinalysis of BC in living mice. Chemical structures of CyP1 and its activated form as CCD in response to APN (R = H or CH_2_CHOHCH_3_) are also represented. Huang et al.: A Renal-Clearable Macromolecular Reporter for Near-Infrared Fluorescence Imaging of Bladder Cancer. Angewandte Chemie. 2020. 59. P. 4416. Copyright Wiley-VCH GmbH. Reproduced with permission [29].

Self-immolative systems (SIS) are systems capable of being activated by a stimulus that will initiate spontaneous intramolecular disassembling, breaking them into their building blocks and, therefore, releasing the drug encapsulated in the structure. This feature has received attention as it is possible to program the drug release according to the specific environment found in diseased tissues such as different pH, reductive conditions, or enzyme expression [28]. The synthesis of a macromolecular system with high renal clearance efficiency and activatable near-infrared fluorescence was reported as a self-immolative system with the potential for real-time noninvasive imaging of orthotopic bladder cancer (Figure 5). The aminopeptidase N enzyme (APN) has paramount importance in the processes of tumor invasion, angiogenesis, and metastasis; accordingly, it is overexpressed in BC, in a way that its levels indicate the tumor size, lymph node metastasis, and metastasis stage. In addition, APN is considered a reliable urinary biomarker for BC detection. However, the challenge related to the optical imaging of BC relies on the ability of probes to go through renal clearance to reach the bladder and to present high specific signals concerning BC-associated biomarkers. The preclinical results of this study showed a renal clearance efficiency of 94% ID 24 h post-injection, and the synthesized macromolecule was effectively able to detect the APN levels related to bladder cancer [29]. 

### 2.4. Adhesion/Bioadhesion/Mucoadhesion

Defined as the formation of an attachment between a biological material and an artificial substrate, bioadhesion is of interest in the development of drug delivery systems. Usually, biopolymers show bioadhesive properties and are used for diverse therapeutic purposes. The bioadhesive polymers can be classified into two groups: (i) specific, with the ability to adhere to certain chemical structures within the biological molecules, and (ii) nonspecific, with the capacity of binding the cell surfaces and the mucosal layer [30].

The direct contact between a delivery vehicle and an absorptive epithelium can result in the intensified specificity and therapeutic effectiveness of delivered compounds [31]. Bioadhesive pharmaceutical formulations are usually designed to enhance drug bioavailability by increasing the residence time of drug compounds and localizing the effect at the targeted site. Simultaneously, they contribute to local drug delivery formulation design, improving bioavailability by avoiding metabolic pathways [32,33].

The current mucoadhesive formulations deal, primarily, with adhesion force-mediated transmucosal drug delivery. Those adhesion forces are generated from the epithelial cell layer, the mucus layer, or a combination of both. There are various mucoadhesive formulations, such as gel, tablet, ointment, powder, and film agent, and their sites of absorption are various mucosal epidermis cells, including buccal and nasal mucosa or ocular surfaces [34].

While mucoadhesive materials can increase contact with a specific site or tissue, the mucoadhesion of a system can be compromised by the natural defenses of the body against the deposition of impurities onto the mucous membrane. Hence, there is a need to possess suitable features to help the maintenance of effective drug concentration at the action site, control the drug release, enable a decrease in drug administration frequency, and increase patient compliance to the therapy [35].

## 3. Mucoadhesive Polymeric Drug Delivery Systems

Mucoadhesive DDS may be of diverse types such as particles with surface modification (cationic or thiolated particulate system) and chemical or physical drug entrapment (system composed of nanoparticles and hydrogels). In a system composed of nanoparticles and hydrogels, it is still possible to obtain non-floating in situ gelations (non-floating composite system of polymeric nanoparticles and hydrogels) [15]. Therefore, to improve drug residence in the urothelial wall of the bladder, after urination, strategic and advanced formulations (Table 1) have been developed through intelligent drug carriers, which combine characteristics of solubility, permeability, adhesion, loading, release, and cytotoxic effect against cancer cells. The mucoadhesion of a system relies on the interaction between the hydrogel and the mucosa structure. The swelling of the hydrogel during contact with the mucosa promotes bioadhesion with physical or chemical interactions and helps to induce cellular uptake. The interactions between the hydrogel and mucosa depend on features such as molecular weight of the polymer, hydration, hydrogen bonding capacity, chain flexibility, charge, and biological environmental factors [36]. In Table 2, some advantages and disadvantages of mucoadhesive DDS are listed. 

An emergent delivery system, based on nanodiamonds (NDs) with a massive surface-to-area ratio, was developed to improve bladder cancer treatment. In this study, chitosan (CH) was used to attribute adhesiveness, increasing the electrostatic force between the positive charge of the polymer and the negative charge of the mucosal wall. Furthermore, the polymer provided steric stability for the colloid, preventing rapid aggregation of the NDs through ion release when in contact with physiological media. To increase the stability of NDs loaded with DOX and coated with CH (CH-NDx) in a neutral pH medium, the CH-NDx were coated with polyanionic molecules (dextran sulfate—DS and pentasodium tripolyphosphate—TPP). Greater release and greater retention of the drug were identified in the TPPCH-NDx formulation (75% and 45%, respectively). TPPCH-NDx also proved to be efficient in the cytotoxic effect against cancer cells, with a lower IC_50_ compared to the IC_50_ of the free drug and the drug trapped only in uncoated NDs. The NDs system proposed by the authors presented encouraging results, highlighting it as a possible option for a more efficient bladder cancer treatment [37].

Studies by Wang et al. [27] focused on the surface modification of mesoporous silica nanoparticles (MSNPs) loaded with DOX, through coating with poly(amidoamine) (PAMAM). In this study, the potential of loading efficiency, release, mucoadhesion, and cytotoxic effect could be controlled by the number of PAMAM dendrimer layers on the surface of MSNPs. Therefore, MSNPs formulations containing zero (G0), one (G1), two (G2), and three (G3) layers of PAMAM were created for investigation. Mucoadhesion assays with mucin particles and in ex vivo porcine bladders showed that the MSNPs-G2 formulation showed greater adhesion to the urothelial mucosa compared to MSNPs-G0. This result is associated with the capacity for electrostatic interaction between positively charged PAMAM and negatively charged urothelium mucin. The loading efficiency and 100 h DOX release for MSNPs-G2 were 95.5% and 65.3% vs. 94.7% and 93.8% for MSNPs-G0, respectively. The presence of PAMAM dendrimer decreased the release rate of DOX, but increased the mucoadhesion of the system, allowing sustained release. The authors also observed that the release of DOX@MSNPs-G2 is responsive to pH, with values of 89.6% (pH 4.5), 67.7% (pH 6.1), and 34.3% (pH 4. 5). The study of cytotoxic effects had an IC_50_ of 1.07 ug/mL for free DOX and 5.63 ug/mL for DOX@MSNPs-G2. The obtention of a formulation with sustained release is a common problem due to the frequent burst behavior of hydrogels. In this research, this problem was fixed by upgrading the functionalities of the materials, such as improvement of mucoadhesion and pH responsiveness.

Xu and coworkers [38] developed mucoadhesive CH nanoparticles modified with mono-benzylic acid (CB) responsive (SSCB) and nonresponsive (CCCB) carriers to intracellular glutathione (GSH) loaded with the gambogic acid drug (GA) activated by ROS (reactive oxygen species). In this study, drug release could be triggered by increased GSH concentration and ROS levels within bladder cancer cells. To efficiently encapsulate hydrophobic charges, CH was modified by mono-benzylic acid, to create hydrophobicity in the amino groups of CH. Part of these amino groups was modified by hydrophobic portions of the benzyl group. In terms of cytotoxic effect against MB49 (mouse bladder cancer cells) and NIH-3T3 (mouse fibroblast cells), the IC_50_ of GA (free drug) was lower than that of GB (drug activated with ROS). For both cells, the best IC_50_ was in the order GA < SSCBGA < CCCBGA. The study of mucoadhesion was carried out in vivo, with fluorescence imaging testing using mice. The tested formulations were responsive (SSCBGB) and non-responsive (CCCBGB) GB, with Cy7.5 as a positive control. After 60 h of incubation, SSCBGB and CCCBGB presented enhanced adhesion when compared to free control. In short, cationic CH promoted the mucoadhesion of the prodrug and ROS-related properties gave SSCBGB the precision to deliver GA to bladder cancer cells. This system presented, thus, a great contribution to bladder treatment studies, as it represents a system with improved residence time and with targeting features. 

Another system, composed of a polyacrylamide nanogel (PAm) functionalized with a cationic amine group (NH_2_) and loaded with the hydrophobic drug docetaxel (DTX), was prepared as a mucoadhesive platform for cancer treatment. The interaction between the hydrophilic matrix and the hydrophobic drug constituted a slow and sustained release of up to 9 days; after 9 days of experimentation, 76% of DTX was released from the PAm-NH2-DTX nanogels. The authors carried out a study of the mucoadhesion of PAm-NH2-DTX nanogels comparing them with free CH. It was identified that PAm-NH2-DTX nanogels were more mucoadhesive than CH. The cell viability in human urothelial carcinoma cell lines, UMCU3 and T24, for PAm-N H2-DTX and PAm-DTX was similar to free DTX, but PAm-NH2-DTX exhibited superior inhibition compared to UMUC3 cells when compared to T24 cells, with a minimal inhibitory concentration (IC_50_) of 5.6 ng/mL versus 535.6 ng/mL, respectively. The IC_50_ difference became even more pronounced as exposure time increased. Thus, this study represented an important option of polymer functionalization to increase mucoadhesive properties, extending the options for systems that present electrostatic interaction with urothelium beyond those containing CH [39].

Studies by Kolawole et al. emphasized the use of CH with β-glycerophosphate (CHGP) for its ability to form a thermosensitive in situ gelling system, with physical crosslinking at 37 °C and pH 6. The study related the thermogelling behavior, drug release, retention, and mucoadhesive properties with the molecular weight of chitosan. In this way, CH of low (LCH), medium (MCH), and high (HCH) molecular weight was used for the formulations. HCH in the presence of GP (HCH-GP) showed a higher gelation index at 37 °C of 138.09 PA, compared to the 59.7 and 30.2 PA of MCH-GP and LCH-GP, respectively. In addition to gelling, mucosal retention was also favored by the high weight of CH. In contrast, formulations with HCH-GP showed less release of MMC, compared to formulations of MCH-GP and LCH-GP; therefore, a higher molecular weight of CH led to the lower release of the drug. The release was still favored in the presence of GP; for all molecular weights of CH, the formulations that contained GP showed greater release of the drug compared to the formulations without GP. In terms of mucoadhesion, the presence of GP in the formulations reduced mucosal adhesion, but the formulations still presented satisfactory adhesion results. Mediating all results, the HCH-GP formulation was identified as promising for intravesical application [40]. In this study, the influence of the molar mass of the polymer chosen on the construction of the system, as well as its behavior concerning mucoadhesion and drug release, was clear.

The mucoadhesion of CH for intravesical administration through the thiolation process has been also investigated and reported in the literature. One of the studies addressed the conjugation of CH glycol (GCH) with thiolated polymers *N*-acetylcysteine (NAC) and glutadione (GSH) to produce microparticles (MP) and nanoparticles (NP). The degree of thiolation by quantifying thiol and disulfide bonds was verified using Ellman’s method. In particular, GSH- and NAC-GC were characterized by 3.6 and 6.3 mmol of immobilized free thiol groups and 0.2 and 0.8 mmol of disulfide bonds per gram of polymer conjugate, respectively. Using the mucin particle method, mucoadhesive properties were measured as a function of turbidimetry and zeta potential in artificial urine with pH 5.0 and 7.0. Conjugates of GC with NAC (NAC-GC) and with GSH (GSH-GC), at both pH, showed greater mucoadhesion compared to unconjugated GC. In all profiles, the NAC-GC formula had greater adhesion strength. Thus, it is possible to observe that the preservation of the thiol groups from oxidation and the greater formation of disulfide bonds resulted in the best mucoadhesion property. Polymer thiolation has been a frequent strategy for mucoadhesion improvement [41]. Moreover, studies conducted by Kolawole et al. (2018) showed that, for CH, mucoadhesion is linked to the degree of methacrylation of H-CH (370 kDa) with anhydrous methacrylate. Different levels of the degree of methacrylation in CH were approached as low (LMe-CH) and high (HMe-CHi) molecular weight. Through the ex vivo study in porcine bladder with sodium fluorescein (FS), the high degree of methacrylation formula revealed better mucoadhesion, due to the presence of a higher percentage of unsaturated methacrylate groups that form covalent bonds with thiols present on the mucosal surface.

The mucoadhesion of high-molecular-weight CH was also evaluated under the influence of the degree of boronation, conjugated by reaction with 4-carboxyphenylboronic. This study pointed out that boron CH can interact with the mucosal surface through three mechanisms: (i) covalent bonding of phenylboronic acid with mucosal sialic acid, (ii) hydrogen bonds with glycoproteins, and (iii) electrostatic interaction between cationic polymer and sialic acid. Therefore, using the traction method, it was possible to observe that the mucoadhesive property was improved in the formulation containing a high degree of boronation (HBChi), due to the greater presence of boronate groups interacting with the mucosal surface. This represents another possibility to confer enhanced mucoadhesion features to polymers by modifying their structures [47].

### 3.1. Theories and Mechanisms

Mucoadhesion could be explained by some theories that include the electronic theory, the wetting theory, the adsorption theory, the diffusion theory, the mechanical theory, the cohesive theory, and the fracture theory [48,49]. 

**Figure 6:**
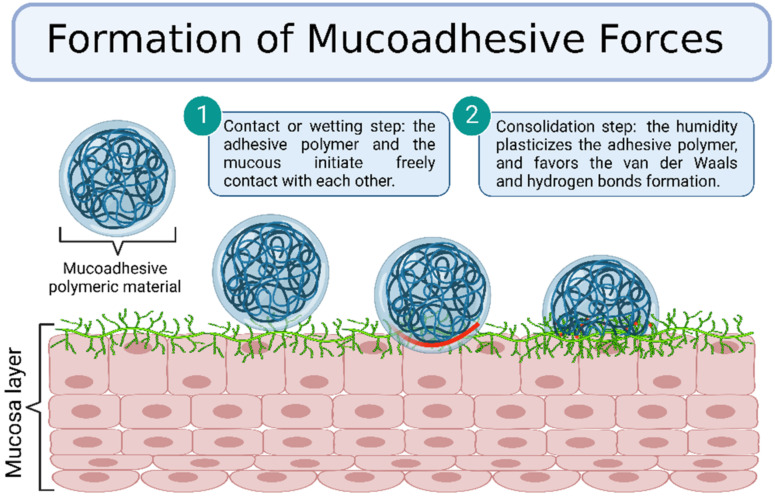
Formation of mucoadhesive forces scheme. Created with BioRender.com Adapted from Biomaterials and Bionanotechnology, Tekade et al., Thiolated-Chitosan: A Novel Mucoadhesive Polymer for Better-Targeted Drug Delivery Muktika, pages 459–493, Copyright (2019), with permission from Elsevier [50].

Despite the complexity of the mechanism responsible for the formation of mucoadhesive forces, there are two general steps involved in the process, namely, contact and consolidation (Figure 6). In the contacting step, the adhesive polymer and the mucous initiate free contact with each other, sometimes influenced by external forces such as the peristaltic movements of the gastrointestinal tract, motions of organic fluids, or Brownian movements. Thus, both attractive and repulsive forces act and the adhesion process initiates only when the repulsive forces are surpassed. This initial step can be correlated to wetting, electronic, adsorption, and mechanical theories [48].

In the consolidation step, the humidity plasticizes the adhesive polymer and favors the formation of van der Waals and hydrogen bonds. Initially, the adhesive polymer and glycoproteins from the mucous interact by interdiffusion and form secondary bonds. After that, the adhesive polymer forms an instantaneous gel hydrated by the aqueous environment [48]. This step is regarded as the diffusion and the cohesive theories, which enforce the idea that the mucoadhesion mechanism is better explained by combining all the mentioned theories [49]. This section describes the various theories and mechanisms involved in the mucoadhesion process.

#### 3.1.1. Electronic Theory

**Figure 7:**
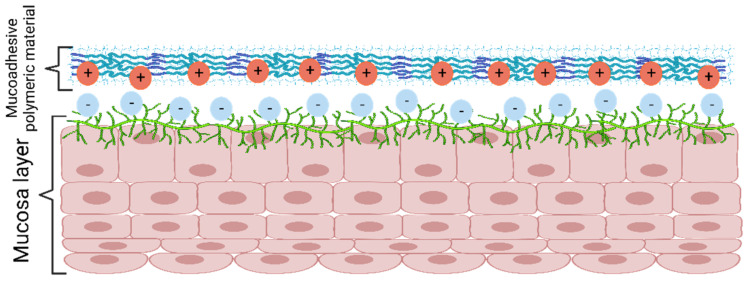
Mucoadhesion mechanism according to electronic theory. Created with BioRender.com Adapted from Biomaterials and Bionanotechnology, Tekade et al., Thiolated-Chitosan: A Novel Mucoadhesive Polymer for Better-Targeted Drug Delivery Muktika, pages 459–493, Copyright (2019), with permission from Elsevier [50].

The electronic theory (Figure 7) explains the presence of attractive forces between the biological and the adhesive system surfaces due to the formation of an electrical double layer produced from the electron transfer among the surfaces [51]. 

#### 3.1.2. Wetting Theory

**Figure 8:**
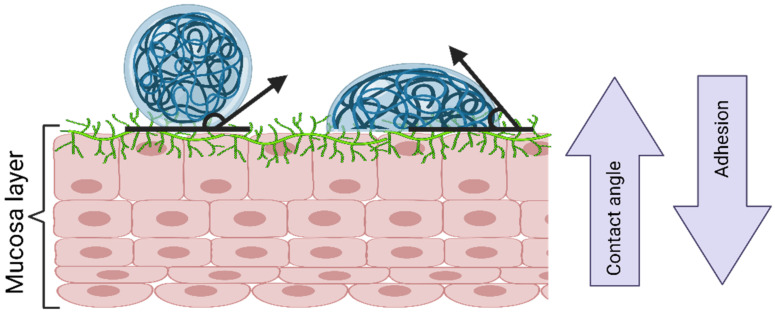
The wetting theory according to contact angle. Created with BioRender.com Adapted from Biomaterials and Bionanotechnology, Tekade et al., Thiolated-Chitosan: A Novel Mucoadhesive Polymer for Better-Targeted Drug Delivery Muktika, pages 459–493, Copyright (2019), with permission from Elsevier [50].

The wetting theory is applied to adhesive systems with low viscosity and high affinity to the substrate. It correlates the adhesion strength to the contact angle of the low-viscosity system (Figure 8), the spreadability coefficient (the difference in the surface energies between the biological surface and the liquid), and the work of adhesion (the energy needed to separate the two phases). In general, at contact angles close to zero, the adhesion strength is benefited due to the increased contact area. In addition, higher individual surface energies are correlated with a better adhesive strength of the interface [49].

#### 3.1.3. Adsorption Theory

The adsorption theory approaches the presence of intermolecular forces, namely, hydrogen bonding and Van der Waals forces, that act between the biological substrate and the adhesive material [51]. Despite the isolated interaction being weak, the combined effect of several forces could lead to strong interactions [48].

#### 3.1.4. Diffusion Theory

**Figure 9:**
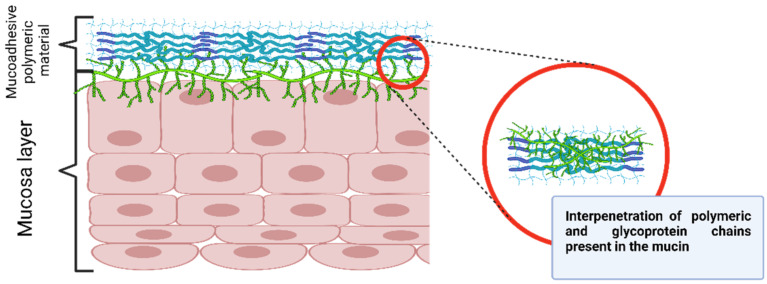
Diffusion theory mechanism. Created with BioRender.com.

The diffusion theory presumes the polymer chain interpenetration through the substrate surface, forming a network structure (Figure 9). The depth of penetration depends on the polymer diffusion coefficient, flexibility and mobility of the mucin structure, the polymer–substrate contact time, the mutual solubility, and the similarity in the chemical structures [51].

#### 3.1.5. Mechanical Theory

The mechanical theory assumes the diffusion of the low-viscosity polymeric system to an irregular and rough biological surface which must increase the surface area available for interaction, forming an interlocked structure that benefits the adhesion process, as well as viscoelastic and plastic dissipation of energies [48].

#### 3.1.6. Cohesive or Fracture Theory

The cohesive theory postulates that the mucoadhesion phenomenon occurs mostly due to the intermolecular interactions among the polymer molecules and biomolecules present in the mucus [52].

This theory considers the strength required for detaching two surfaces after adhesion. Therefore, the adhesion is described by the force required for rupture, in addition to the factors that promote the adhesive interaction, considering the other theories to explain the mucoadhesion process. Moreover, the rupture of the adhesive bonds occurs through the interface, mostly at the weakest point of interaction between the surfaces. This theory is mainly applicable to rigid and semirigid bioadhesive materials [53,54].

Based on previous theories, mucoadhesion can be generally classified into two categories: (i) chemical, which comprehends the electronic and the adsorption theories, and (ii) physical, which includes the wetting, diffusion, and cohesive theories. 

### 3.2. Methods to Evaluate Mucoadhesivity of Polymers

There are several in vivo, ex vivo, and in vitro methods for assessing the efficacy of the mucoadhesion capacity of a polymer system. In vitro tests are firstly performed to screen potential bioadhesives and are usually accomplished using mechanical and rheological tests. Some typical tests include tensile strength, shear strength, rheological methods, colloidal gold staining method, mechanical spectroscopic method, and falling liquid film method. This section describes the tests mentioned above.

#### 3.2.1. In Vitro and Ex Vivo Methods

##### Tensile Strength

This method measures the force required to break the adhesive between the mucoadhesive polymer system and the mucous using testers and balances [48]. For example, Ferreira and colleagues [55] evaluated their adhesive formulations using a texture analyzer.

##### Shear Strength

The shear strength is determined by measuring the required force for a mucoadhesive polymer system to slide from the mucous surface considering the parallel direction to the plane of the contact area. For instance, Silva and collaborators [56] evaluated the synergism between their polymeric adhesive system and mucin using a controlled stress rheometer. 

##### Rheological Methods

The viscometrical method is used to measure the mucin–mucoadhesive polymer bond strength. Thus, the force of mucoadhesion is measured concerning the rheological changes of the polymer–mucin mixtures. The mucoadhesive polymers/mucin mixtures tend to present a higher viscosity than the sum of viscosities of the individual components of the mixture. The interaction between the mucin and the mucoadhesive polymer commonly generates improved viscosity depending on the polymer applied to the system [48].

##### Washability Test 

The washability test is a modification of the Franz diffusion cell to concurrently measure the drug released by diffusion from a mucoadhesive polymeric system and the amount washed by a tangential flow. This method adopts the use of a modified donor that has the chamber closed with two sideways that enable a thermostatic buffer to stream over the sample. The buffer simulates the physiologic conditions and is fluxed over the mucoadhesive polymeric system at a constant rate, being gathered in a beaker under constant stirring. Moreover, the tracer or drug washed is quantified by a proper analytical method [56].

##### Colloidal Gold Staining Method

The colloidal gold staining method is based on the measurement of the change in the color intensity of the mucin molecules promoted by the red colloidal gold particles resulting from the interaction between the mucoadhesive polymer system and the mucin gold conjugates, which tends to develop a red color on the mucoadhesive surface [48]. Huang and colleagues [57] improved the classic colloidal gold staining technique developed by Park in 1989 by developing the Pt-staining method based on test strips to create platinum nanoshells on the surface of colloidal gold. This method not only retains the original advantages of colloidal gold with easy synthesis and bonding but inserts Pt. nanoparticles with excellent catalytic activity as a signal marker to reach sensitive quantitative detection [57,58].

##### Mechanical Spectroscopic Method 

The mechanical spectroscopic method consists of the study of the effect of pH and polymer chain length over mucoadhesion. The difference between the storage modulus of the mucoadhesive polymer system and the individual components at the same concentration is evidenced by the magnitude of the interaction between the polymer and mucin. A higher difference indicates a stronger presumed interaction [48]. 

##### Falling Liquid Film Method

The falling liquid film method is an ex vivo test proposed by Teng and Ho who set small intestine sections from rats on an inclined Tygon tube flute (angle of 45°) [59]. The particle suspension migrates over the mucous interface, and the adhesion strength is determined by the particle portion adhered to the mucous surface [48]. Efiana and collaborators adapted the method for porcine intestinal mucosa, and Sudan Red G was used as a label signal for absorbance measurement [60].

##### Biacore System

The Biacore is an instrument based on the principle of an optical phenomenon, namely, surface plasmon resonance (SPR), which expresses a response of the measurement of the refractive index that varies as a function of the solute content present in a solution that contacts the sensor chip. During the detection process, the polymer molecules are retained on the sensor chip surface, and the mucin suspension migrates through the sensor chip. When the analyte, mucin particle, links to the ligand molecule, the polymer on the sensor chip surface, the solute concentration, and the refractive index on the surface change, increasing the resonance unit response. Once they dissociate, the resonance unit response falls [30,60]. 

##### Confocal Laser Scanning Microscopy (CLSM) Method

Confocal laser scanning microscopy (CLSM or LSCM) is a high-resolution and high-selectivity technique. The main aspect of this optical imaging technique is the possibility of obtaining in-focus images from selected depths. CLSM combines the laser scanning method with the 3D detection of biological objects marked with fluorescent signalers. For example, Dyawanapelly and collaborators [61] evaluated the mucoadhesion properties of the CH oligosaccharide surface-modified polymer nanoparticles developed for mucosal delivery of proteins. 

#### 3.2.2. In Vivo Methods

In vivo mucoadhesion evaluation is generally based on residence time or relative bioavailability assays. Due to the cost, time, and ethical concerns, there are few studies described in the literature. Accordingly, these techniques are sparse in comparison with in vitro and ex vivo methods. In vivo methods are a greater indicator of clinical performance, although they cannot distinguish between mucoadhesion and other factors that influence the residence time besides commonly present high standard deviations [51].

The in vivo data can usually be correlated to in vitro analysis. Low in vitro/in vivo correlation of the mucoadhesive strength indicates that a polymeric system with strong mucoadhesion properties in vitro might not reach longer mucosal residence in vivo, which may be explained by the different environments in vitro and in vivo [51]. Gamma scintigraphy is a technique used for the diagnosis of diseases in neurology, oncology, and cardiology based on the use of gamma-ray emitting radioisotopes that allow following in real time the path of the labeled formulation in the body after administration. Therefore, a formulation under investigation may be tracked inside the body via a gamma-emitting radionuclide label. Moreover, it is also possible to obtain quantitative information about the molecules in organs by counting the radioactivity in them. The radiolabeling of the location/organ of interest takes place by using a short-lived radioisotope that can emit gamma rays, such as ^99^Tc [62].

### 3.3. Mucoadhesive Polymers Suitable for the Development of DDs for Bladder Cancer Treatment

Several different polymers have been used to receive materials suitable for the treatment of bladder cancer. According to the origin of the macromolecules, they could be synthetic (poly(*N*-vinyl-2-pyrrolidone), poly(vinyl alcohol), poly(2-hydroxyethyl methacrylate), Carbopol, Pluronic^®^, etc.) or natural (gellan gum, sodium alginate, hydroxypropyl cellulose, or carboxymethylcellulose). Below, general information on the selected polymers can be found.

Poly(*N*-vinyl-2-pyrrolidone) (PVP) was developed in 1939 by Walter Reppe at BASF. Subsequently, it started being utilized in a multiplicity of sectors: pharmaceutical, cosmetic, and detergent industries. PVP is now being used in several technical applications such as membranes, glue sticks, hot-melt adhesives, and crop protection. Due to its versatile features (such as water solubility, film and complex formation, and adhesive and bonding power), as well as its toxicological harmlessness, PVP is one of the most interesting technical specialty polymers in the field of chemistry [63]. Grant and colleagues recently prepared electrospun nanofibrous mats of CS and PVP for the delivery of the chemotherapy drug 5-fluorouracil (5-Fu) to treat lung cancer. The developed material demonstrated efficiency in killing cells for over 24 h and, therefore, presented potential as a DDS for the application proposed [64]. Nanocomposites of PVP combined with alginate and polydopamine were also prepared and studied regarding their potential for cancer treatment purposes. The release studies showed the capacity of delivering the DOX drug for 50 h and the possibility to combine a photothermal treatment with chemotherapy. The results confirmed the efficacy of the combined therapy, lowering the cell activity to only 13.2% [65].

Poly(vinyl alcohol) (PVA) is a water-soluble, biodegradable (under both aerobic and anaerobic conditions) [66], and biocompatible polymer that is obtained from poly(vinyl acetate) through alkaline hydrolysis. It presents a high ability to form films, with high surface stabilization and chelation properties [67]. This polymer is one of the most important synthetic polymers used in commercial, industrial, medical, and nutraceutical applications [68]. Furthermore, several studies have used PVA in the development of improved cancer therapies [67]. Ullah and coauthors prepared formulations composed of carboxymethyl chitosan and PVA for the delivery of oxaliplatin in the treatment of colorectal cancer. In their studies, they were able to develop a pH-responsive hydrogel which, together with the concentration variation of both polymers, allowed tailoring the delivery properties of the material [69].

Poly(2-hydroxyethyl methacrylate) (PHEMA) is a stable, optically transparent, hydrophilic methacrylate polymer. In the dry state, the material is hard and glassy; however, in polar media, the pendant hydroxyethyl group can extend outward, and the material becomes soft and flexible. Due to its good biocompatibility, PHEMA has been extensively researched for biomedical applications [70], such as hydrogel systems for drug delivery or scaffolds for tissue engineering. PHEMA-based hydrogels can be engineered to possess similar water content and mechanical properties as tissue and exhibit excellent cytocompatibility. The most prominent example of a biomedical device based on pHEMA may be the very first modern soft contact lenses developed by Otto Wichterle around 1960 [71]. 

Carbopol (Carbomer) is a high-molecular-weight, acrylic acid-based polymer crosslinked with allyl sucrose or allyl pentaerythritol that contains between 56% and 68% *w*/*w* carboxylic acid groups [72]. Carbopol polymers were first described and patented in 1957 [73]. Since then, several release tablet formulations, which involve carbomer matrices, have been patented [74]. Today, Carbopol polymers are widely accepted ingredients in pharmaceutical dosage systems of almost every form, from controlled-release tablets to oral suspensions and other novel delivery systems, as well as a variety of topical products. Carbomers demonstrate good mucoadhesion, particularly at low pH values where they are present in a protonated state [75]. Carbopol 940 has been combined with micelles containing paclitaxel for the local treatment of melanoma. The studies showed that the formulation proposed was capable of increasing the retention the permeability of the drug into the skin. One of the reasons for this behavior was the positive charges presented in the polymer that were also helpful to promote melanoma cellular uptake and improve the in vitro cytotoxicity [76].

Pluronic^®^ (Poloxamer) is a synthetic amphiphilic copolymer based on hydrophilic poly(ethylene oxide) (PEO) blocks and hydrophobic poly(propylene oxide) (PPO) blocks organized in a triblock structure PEO–PPO–PEO. PEG refers to polyols of Mw below 20,000 Da, while PEO is relevant to polyols with higher molecular weight [77]. The properties of the Pluronic^®^ copolymers can be changed by adjusting the molar mass ratio between the PEO and PPO blocks [78]. In an aqueous environment, these block copolymers self-assemble into micelles with a hydrophilic PEO outer shell that interfaces with water. Since these micelles are amphiphilic, they could accommodate lipophilic molecules in the central hydrophobic core area. Consequently, Pluronic^®^ micelles are effectively used as drug carriers because their assemblies can act as passive drug containers [79]. Researchers have developed a system composed of chitosan thioglycolic acid nanoparticles loaded with gemcitabine HCl and dispersed into a bioadhesive CH gel or in an in situ gelling poloxamer solution as potential formulations for the treatment of superficial bladder cancer. Both formulations presented mucoadhesive properties and the capacity of enhancing drug residence time; however, poloxamer lost its gelling property when diluted in artificial urine at body temperature. Thus, it would be recommended to empty the bladder of the patient before application of this formulation to guarantee its gelling capacity and, therefore, good mucoadhesion and a sustained release [80].

### 3.4. In Situ Gelling Polymers

In situ gelling systems are polymeric formulations in solution before entering the body, where, under physiological conditions, they change into a gel form. This can occur through different types of devices correlated with the properties of the polymers used in the delivery system and due to physical or chemical crosslinking that can be triggered by factors such as changes in temperature, pH, and the presence of ions. The sol-gel transition is very common for thermosetting polymers, i.e., those that present an upper-critical solution temperature or lower-critical solution temperature, in which, according to temperature changes, the gel-forming units interact with each other via physical (van der Waals and electrostatic) or covalent bonds, forming a gel network. These systems are characterized by being easy to administer, presenting the sustained release of the drug at the target, with the possibility to be administered via different routes to obtain local or systemic effects of the loaded drug [81,82].

The pH-specific polymers have the characteristics of structures with ionizable groups—weakly basic or acidic. Changing the pH will produce changes in the ionization state, as well as in the solubility and conformation that result in polymer gelation [83].

Thermosensitive in situ gelling systems have sol-gel transition triggered at temperatures close to physiological ones (32–37 °C). This transition occurs via a change in the aqueous solubility of the polymers, characterized by structures of hydrophobic and hydrophilic groups. With the increase in temperature, there is a rearrangement of polymer–water interactions, which is responsible for the polymer separation and dehydration of the solvated polymer chains in a rapid way. These polymers that have hydrophobic and hydrophilic segments form self-assembled micelles that, at higher temperatures, cause their packing and, thus, the change of the solution into gel form [84]. Some polymers are sensitive to ions such as alginate, gellan gum, and pectin, and crosslinking occurs due to some monovalent or divalent cations that are present in physiological fluids, such as tears and saliva. The viscosity of the gel obtained depends on the cation type and its concentration [85].

#### 3.4.1. Thermo-Responsive Systems 

**Figure 10:**
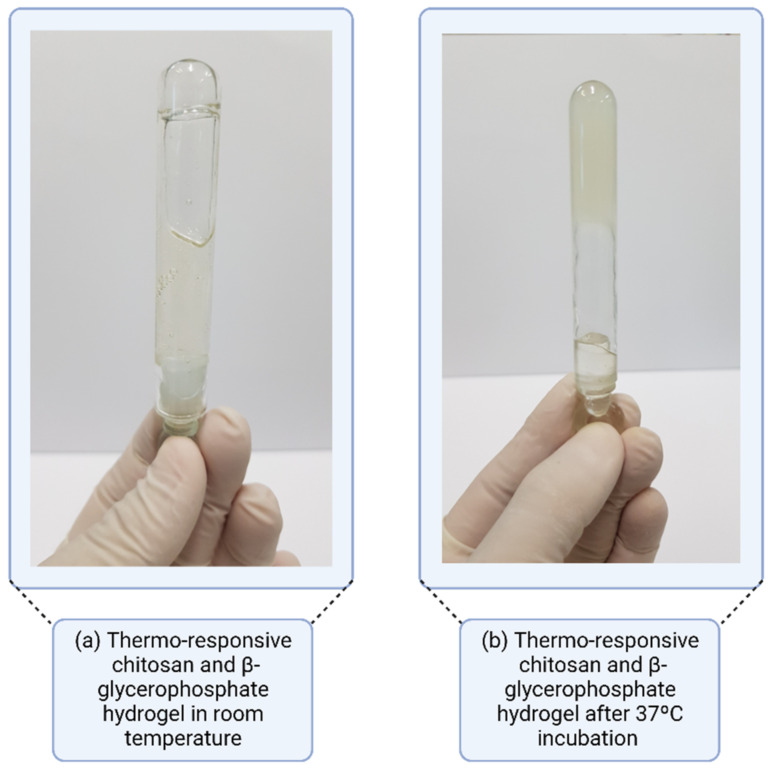
Thermo-responsive gel system with lowest critical temperature.

In situ gelation triggered by temperature occurs in thermo-responsive polymers, with sol-gel transitions directly linked to specific temperature limits. When a polymer solidification occurs above a temperature limit, the system presents a “lowest critical temperature” [86]. Thus, it is understood that, at low or room temperatures (20–25 °C), these thermo-responsive polymers have a fluid aspect, whereas, under physiological conditions with a temperature between 35–37 °C, they present a gel behavior (Figure 10). These gels sensitive to in situ temperature have high fluidity and low viscosity, providing easy application [87]. Solutions that show a sol-gel change when cooling present a “higher critical temperature” due to micellar growth, hydrophobic interaction, and transition from the coil to a helix. This phenomenon is observed in gelatin or carrageenan solutions, where a random coil shape occurs in the solution, thus generating a continuous network via partial helix formation after cooling [88].

Some polymers present gelation at a higher temperature, due to the gradual loss of water when the temperature rises, thus increasing the intermolecular interaction and aggregation of the network structure, leading to the gelation of the system [89]. For example, hydroxypropylmethylcellulose presents the lowest critical temperature of the solution between 75 and 90 °C, while methylcellulose presents it at 40 to 50 °C [90].

#### 3.4.2. pH-Responsive Systems 

The pH is considered an important factor governing the degree of ionization of polymeric systems and their solubility in water. Repulsive electrostatic forces and osmotic forces in the presence of ions cause the polymer to swell pH-dependently or to disintegrate the gel [91].

Some polymers undergo gelling triggered by changes in pH, based on ionizing proportions. Polymers such as polyacrylic acid and carbopols have high molecular weight and a large number of carboxylic acid groups. These polymers show little swelling at low pH, as they have low concentrations of dissociated acid portions. When there is an increase in pH, an electrostatic repulsion triggered by additional charges leads to an expansion of these polymers, promoting their gelation [92]. If acidic pH is proposed to develop in situ gelling formulations using these polymers to maintain low viscosity, the stability could be affected, especially in pH-sensitive drugs [86].

Another polymer that has pH-sensitive properties is CH, which is a polymer rich in amine bonds. In acidic pH, it is soluble due to electrostatic repulsion, whereas, at pH above 6.2, it forms a dissociated precipitate [92]. Studies with a CH derivative containing palmitic acid linkages in its free amines (*N*-palmitoyl CH) showed gelling properties at physiological pH. This allowed applying the liquid solution at pH 6.5, which became solid after reaching pH 7.4 [93]. At low pH, free amines are protonated, and electrostatic forces block the cohesive attraction between polymer chains; when there is an increase in pH, the hydrophobic interactions between palmitoyl groups are more significant, boosting the condensation of molecules of *N*-palmitoyl CH.

#### 3.4.3. Ionic-Responsive Systems 

In situ gelation triggered by ions occurs through the interaction of anionic fractions present in the molecule and cations, such as calcium (Ca^2+^), usually present in body fluids such as vaginal, nasal, or lacrimal [94].

**Figure 11:**
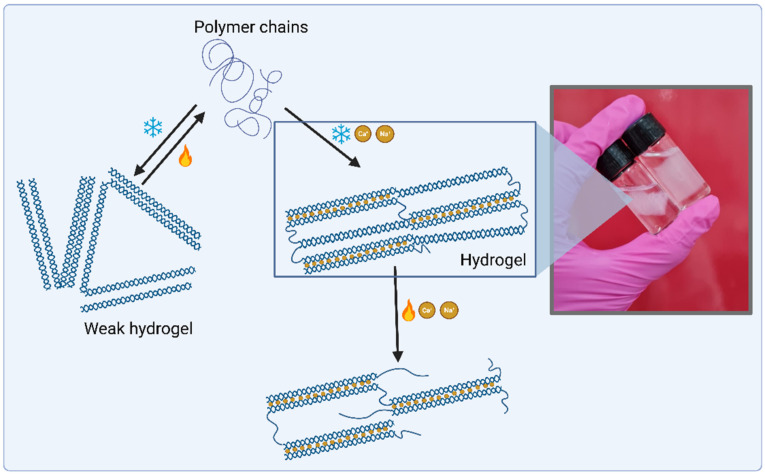
Gellan gum hydrogel system triggered by the presence of cations and temperature. Above 50 °C, gellan gum polymer chains are disordered, but the colling process of the solution induces the formation of double helices stabled by hydrogen bonds. The presence of cations in the solution allows the interconnection of these helixes and the formation of a 3D matrix. Created with BioRender.com.

Ionic-sensitive polymers commonly used for in situ gelling preparation are natural polysaccharides such as alginate, gellan gum (Figure 11), and pectin, which are generated by gels triggered by the interaction between carboxylic acid residues present in the polymeric structure and surrounding cations. Monovalent cations weaken the electrostatic repulsion, promoting hydrophobic interactions, while divalent cations cause the association of helical sections of polymer chains, generating junction zones. It should be noted that the variation in ion concentration can lead to different mechanical properties, creating an ‘egg box’ structure responsible for gelling, resulting in heterogeneous gels [95].

#### 3.4.4. In Situ Gelation Triggered by Genipin

Less toxic alternatives than glutaraldehyde for polymer crosslinking have been studied, and genipin, a compound of natural origin, was demonstrated to be a promising alternative. The pH of the mixture influences the reaction mechanism, giving genipin a ring-opening polymerization under basic pH conditions, while a Schiff reaction together with primary amines gives rise to the formation of crosslinked networks under acidic or neutral conditions. In these cases, a nucleophilic attack occurs on genipin, resulting in the formation of heterocyclic amines and, thus, crosslinking via genipin bounds [96]. Researchers investigated the in situ gelation of the collagen–genipin mixture in treatments for gastrointestinal ulcers, with good results under physiological conditions [97].

### 3.5. Rheological Aspects

Rheology is the study of the material flow and its deformation behavior, which can be measured by applying force to a sample. Combined with formulation viscosity, plasticity, and elasticity, the rheological behavior may impact product manufacturing, long-term stability, appearance, dispensing, sensory properties, packaging, and in vivo performance [98].

One essential property of semisolids and viscoelastic materials is the rheological behavior. Gels can be cited as a typical example of a pharmaceutical semisolid that behaves in a non-Newtonian manner. The viscosity of a gel is defined as a flow curve reflecting the shear stress as a function of shear rate or strain, and the viscoelastic properties are presented as a frequency sweep reflecting the moduli at increasing frequencies [99].

Mathematical modeling can be applied in rheology to reliably predict the rheological properties of concentrated or diluted polymeric liquids. With those models applied in the assessment of hydrogel networks and their rheological characteristics, it is possible to identify key parameters for the process, formulation, and mechanisms of drug delivery. Consequently, the mathematical understanding of the gel-forming material properties and of the way the formulation and process parameters interact can facilitate the intelligent design of a hydrogel network [100].

The understanding of the rheological properties of hydrogels is key to gaining insight into the mechanical properties, viscoelastic behavior, and interactions between the hydrogel components. Those properties are the fusion of multiple factors such as the structure and nature of polymers, temperature, ionic strength, pH, concentration, and crosslinking of polymers components within the hydrogel. Further knowledge about those properties helps the determination of possible industrial applications for the synthesized hydrogel [101].

A thorough rheological analysis helps in understanding the properties of any synthesized material. The rheological study helps in perceiving the viscosity, elasticity, crosslinking, flow, and mechanical behavior of the material, in response to an applied strain or stress. Those kinds of properties are also known to vary with changes in the molecular network, and they play a crucial role in the determination of the field of application of the synthesized material [102].

When it comes to developing formulations for intravesical applications, rheology is a key factor to allow instillation via a catheter and to understand its behavior inside the bladder concerning bioadhesion, stability, and drug release. 

## 4. Conclusions and Future Perspectives

Bladder cancer’s survival rate is high when compared to other types of tumors such as pancreatic and glioblastoma. However, it affects considerably the quality of life of the patients. Additionally, many of them present the progression of superficial cancer, pass through chemoresistance, and end up developing muscle-invasive bladder cancer [8]. Depending on the tumor stage, treatment may englobe intravesical chemotherapy, which usually demands frequent catheter insertions due to the reduced residence time of the drug. Several procedures of chemotherapy instillations may irritate the urinary tract and other side-effects, which have encouraged researchers to develop drug delivery systems for bladder cancer treatment. 

Drug delivery systems must present some important properties such as specific release at the lesion tissue or targeted cells and biocompatibility. Concerning nanotechnologies, they may be very helpful in targeting nonspecific drug release, via passive and active approaches, as an option to solve side-effects related to chemotherapy [8]. 

Bioadhesion is an important strategy to enhance drug residence time and target delivery. Among other key points to evaluate, mucosal thickness, low absorptive surface area, mucosal microbiome, and mucosal secretion are challenges that must be taken into account when developing a mucoadhesive system with optimum therapeutic response [103]. Natural and synthetic polymers have been explored due to their properties of mucoadhesion with different mechanisms as possible vehicles for drug delivery in bladder cancer treatment. However, most of the research in progress is still in need of further in vitro and in vivo studies. Moreover, therapeutic outcomes and topics concerning safety must be evaluated by clinical trials, as in vitro and in vivo results provide limited information. The progress in these aspects of new and sophisticated drug delivery systems will allow the improvement of bladder cancer treatment [104].

## Figures and Tables

**Table 1:** Mucoadhesive polymeric systems for controlled drug release in the bladder—advanced formulations.

Drug	Carrier	Polymer	Cancer Cells	Encapsulation Efficiency (%)	Reference
Doxorubicin	Nanodiamonds with surface modification	Chitosan	HT-1197	>90	[37]
Doxorubicin	Nanoparticles with surface modification	Poly(amidoamine)	UMUC3	>90	[27]
Gambogic acid	Nanoparticles with surface modification	Chitosan	MB49 and MH-3T3	-	[38]
Docetaxel	Nanogel	Polyacrylamide	UMC3 and T24	>90	[39]
MMC	Gel	Chitosan/β-glycerophosphate	-	-	[40]
Fluorescein diacetate	Micro and nanoparticles	CH glycol (GCH), *N*-acetylcysteine (NAC), and glutadione (GSH)	-	12.2–100%	[41]
Gemcitabine hydrochloride	Microspheres	Carbopol 2020 NF, Eudragit E100 (EE100), poloxamer and chitosan	T24 (ATCC HTB4TM) and RT4 (ATCC HTB2TM)	>80	[42]
Paclitaxel	Nanoparticles	Gelatin	-	0.52	[43]
Doxorubicin and peptide-modified cisplatin	Nanocapsules	Chitosan, polymethacrylic acid	UMUC3	>80	[44]
Paclitaxel	Liposomes in a gel system	Gellan gum	NBT-II and T24 (ATCC, USA)	>90	[45]

**Table 2:** Mucoadhesive DDS: advantages and disadvantages [46].

Advantages	Disadvantages
Prolong drug residence time at the tumor site	Dislodgement of the formulation may happen
Increase drug bioavailability	Overhydration may compromise the formulation structure
Reduce dosing frequency	
Improve drug permeability	
Reduce the dose of drug administered	
Fast onset of action	

## Data Availability

Not applicable.
